# Evolution of Retinal Neuron Fractality When Interfacing with Carbon Nanotube Electrodes

**DOI:** 10.3390/bioengineering11080823

**Published:** 2024-08-12

**Authors:** Aiden P. Dillon, Saba Moslehi, Bret Brouse, Saumya Keremane, Sam Philliber, Willem Griffiths, Conor Rowland, Julian H. Smith, Richard P. Taylor

**Affiliations:** 1 Department of Physics, University of Oregon, Eugene, OR 97403, USA; 2 Materials Science Institute, University of Oregon, Eugene, OR 97403, USA; 3 Department of Biology, University of Oregon, Eugene, OR 97403, USA; 4 Department of Biology, Institute of Neurobiology, University of Oregon, Eugene, OR 97403, USA

**Keywords:** biocompatibility, bio-inspiration, electronics, fractals, implants, neurons

## Abstract

Exploring how neurons in the mammalian body interact with the artificial interface of implants can be used to learn about fundamental cell behavior and to refine medical applications. For fundamental and applied research, it is crucial to determine the conditions that encourage neurons to maintain their natural behavior during interactions with non-natural interfaces. Our previous investigations quantified the deterioration of neuronal connectivity when their dendrites deviate from their natural fractal geometry. Fractal resonance proposes that neurons will exhibit enhanced connectivity if an implant’s electrode geometry is matched to the fractal geometry of the neurons. Here, we use in vitro imaging to quantify the fractal geometry of mouse retinal neurons and show that they change during interaction with the electrode. Our results demonstrate that it is crucial to understand these changes in the fractal properties of neurons for fractal resonance to be effective in the in vivo mammalian system.

## 1. Introduction

Miniaturization of electronic chips offers surgeons the possibility to implant devices in the human body to provide neural stimulation. As a result, over 160,000 deep-brain stimulation implant surgeries have been conducted to treat neurological disorders, with an estimated 12,000 new surgeries performed annually [1]. Devices have also been implanted into retinas with the aim of restoring vision to patients experiencing degenerative retinal diseases [2,3,4,5,6,7,8,9,10,11]. Beyond improving the performance of implantable medical devices, studying neuron responses to implants offers a unique opportunity to investigate the fundamental behavior of neurons—and consequently, the extent to which this behavior can be controlled. While neurons maintain responsibility for transporting the body’s electrical signals, glial cells provide crucial life support for the neurons. Consequently, due to their roles in controlling the neural network’s structure and functionality, the interactions of glia with implants are also important [12,13,14,15]. 

Most implants feature electrodes that either stimulate or sense local electrical signals as they pass through the neuronal networks [16], while also acting as physical scaffolds for cell growth. The biocompatibility of these implants is ensured by three key factors: chemical environment, physical texture, and electrode geometry. We focus on the latter, which has been the least studied factor, with a specific exploration of bio-inspired electrode designs. Neurons are described by fractal geometry owing to their dendritic branches that repeat over increasingly fine size scales. In contrast, typical implants are based on Euclidean geometry built off shapes such as lines, squares, and grids. This presents a fundamental mismatch between the geometries of biological cell systems and the implants used to interact with them. Thus, a rigorous understanding of the natural geometry of neuronal networks provides a clear path towards increasing the biocompatibility of future implants.

In previous work, we have demonstrated that the fractal dimension, *D*, of neuron arbors serves as an effective metric for measuring the ratio of fine- to coarse-scale dendritic patterns in the arbor [17,18,19,20]. A neuron’s *D* value quantifies its ability to connect to its neighbors by tuning the physical profile of its dendrites [17,18,19,20]. Healthy neurons tend to cluster around a *D* value that yields a ‘balanced connectivity,’ optimizing the trade-off between connection capabilities and the associated cost constraints of establishing connections [17,18,19,20]. This research builds on Ramon y Cajal’s wiring economy principle, which proposes that neurons minimize wiring costs [21] such as metabolic expenditures and the mass resource required to form the neuron. Notably, *D* has been shown to effectively capture this functional optimization despite dendrites’ fractal-scaling behavior occurring over a relatively narrow range of size scales (typically less than two orders of magnitude) [17,18,19,20]. 

Mathematical models have been employed to study neurons as they deviate away from their natural *D* value by, for example, straightening out or curling up their dendrites [17,18,19,20]. Measuring the resulting deterioration in balanced connectivity led to the ‘fractal resonance’ hypothesis, informing bio-inspired electrode designs. Fractal resonance proposes that electrode patterns that match their *D* value to that of the neurons they interact with will encourage natural growth on the electrodes, benefitting from the favorable functions associated with being tuned to the ‘natural’ geometry of the neurons. In principle, resonance could be achieved through a two-step process: (1) measuring the *D* values of neurons as they grow in an unrestricted environment and (2) growing neurons on electrodes with *D* values that match those of the unrestricted dendrite growth. 

In this paper, we present a preliminary analysis for the first step while highlighting some of the associated challenges, with our initial research employing the simple case of two-dimensional planes as platforms for unrestricted growth. We have imaged cultured mouse retinal neurons on the surfaces of multi-walled, vertically aligned carbon nanotubes (VACNTs). Since the surface texture of VACNT electrodes exhibits a height roughness significantly smaller than the lateral distances across the plane (less than one micrometer in comparison to several millimeters, respectively), VACNTs effectively serve as two-dimensional planes. Although the dendrites growing on these electrodes are restricted to the two-dimensional plane, they grow physically unrestricted across it. 

Building on previous CNT compatibility studies [16,22,23,24,25], we conduct in vitro experiments using postnatal day 4 (PN4) mouse primary retinal cells to study the unrestricted growth of neurons in addition to cell–electrode interactions. The cultures are studied as they evolve over 17 days in vitro (DIV) by utilizing fluorescence imaging. By measuring the neurons’ *D* values at 7 DIV and 17 DIV, we show that their fractal geometry evolves during the process of network formation while interacting with the VACNT surface. Accordingly, this complicates the application of the fractal resonance principle—with a non-static neuron fractal geometry, there is not a unique *D* value to match to the electrode. Later, we discuss the broader implications for implants designed to interface with diseased neurons, such as retinal bipolar cells in eyes undergoing macular degeneration.

## 2. Background

A broad range of experiments have investigated the impact of different patterned surfaces on the growth behavior of neuron dendrites and axons [26]. Patterning techniques have varied from curving the surface to introducing shaped regions of the surface texture [27,28]. Examples of different surface textures included parallel grooves [29,30,31,32,33] and zig-zag ‘micro-tracks’ [34,35,36]. While the neurons preferred the direction of minimum curvature [27,28], the directional guidance induced by patterned textures depended on the width of the pattern, the depth of the texture [29,30,31,32,33,34,35,36], the neuron subtypes, and their stage of development. Throughout this broad range of experiments, there is a primary focus on investigating the manipulation of neuron growth patterns through the interaction with artificially patterned surfaces. Instead, our bio-inspired approach investigates the conditions that encourage neurons to grow according to their natural behavior. Our previous experiments on zig-zag micro-tracks guided dendrites around large corner angles [35]. Interestingly, a similar experiment found that neurons grew longer processes when guided by zig-zags in comparison to straight tracks [34]. Building off the speculation that the added complexity accompanying zig-zag corners acts as a crude initial step towards mimicking a neuron’s natural weave pattern, the next logical step is to incorporate more corners across multiple size scales—resulting in the generation of a fractal electrode. More concretely, this simple thought experiment aligns with our previous neuron modelling research, which quantifies the increase in a neuron’s connectivity as it evolves from having straightened dendrites to having a natural fractal weave [14,15,16,17]. In this, it is evident that the fractal resonance hypothesis marks a shift from biocompatibility to biophilia. More than simply tolerating the introduction of artificial electrodes, we aim for neurons to be attracted to and thrive on bio-inspired ones.

In our experiments, we employ carbon nanotubes (CNTs) as the electrode material [22]. CNTs have been shown to promote neuronal adhesion and growth [37,38,39] due to their surface texture and chemical composition. Notably, their nano-scale roughness has been proposed to mimic the properties of the extracellular matrix (ECM) [40,41,42]. The mechanical flexibility of CNTs is another attractive feature, as neurons are known to adhere to and grow on softer substrates [43,44]. This flexibility, along with the texture of our pristine CNTs, is sufficient for biocompatibility with a neuronal network [22]. Previous studies also demonstrate that electrically biased CNT electrodes stimulate [45,46] and boost neuron signal transmission [47,48], indicating the remarkable potential for future electrical applications.

We will use VACNTs that grow vertically from deposited metal catalysts on a silicon dioxide (SiO_2_) substrate (see Methods). On average, they grow to heights of approximately 25 μm. This height aids penetration into the neural tissue in implanted electrodes [49]. In this current study, we utilize square-shaped regions of the VACNTs with side lengths of 2.145 mm. As they grow, they tangle into a conducting ‘forest’ featuring a top surface (or canopy) with the necessary texture to support an abundance of healthy neurons. The comparatively smooth SiO_2_ regions surrounding the VACNT textured square regions are known to result in an accumulation of glial cells, providing life support for the neurons cultured on the VACNTs [44,50,51]. In previous experiments, this difference in cell responses to different surfaces was exploited specifically to ‘herd’ neurons and glia to different regions. Neurons largely adhered to the textured VACNT regions, while glial cells primarily covered the smooth SiO_2_ regions [16,52,53]. This allowed neurons to grow interruption-free of neuron–electrode interactions while maintaining enough proximity to the glia to benefit from their trophic and metabolic support, which is necessary for neuron health and operation (namely, the transmission of electrical signals). Meanwhile, the VACNT electrodes act as the physical scaffold, ensuring neuron-rich electrodes that maximize stimulation. 

**Figure 1:**
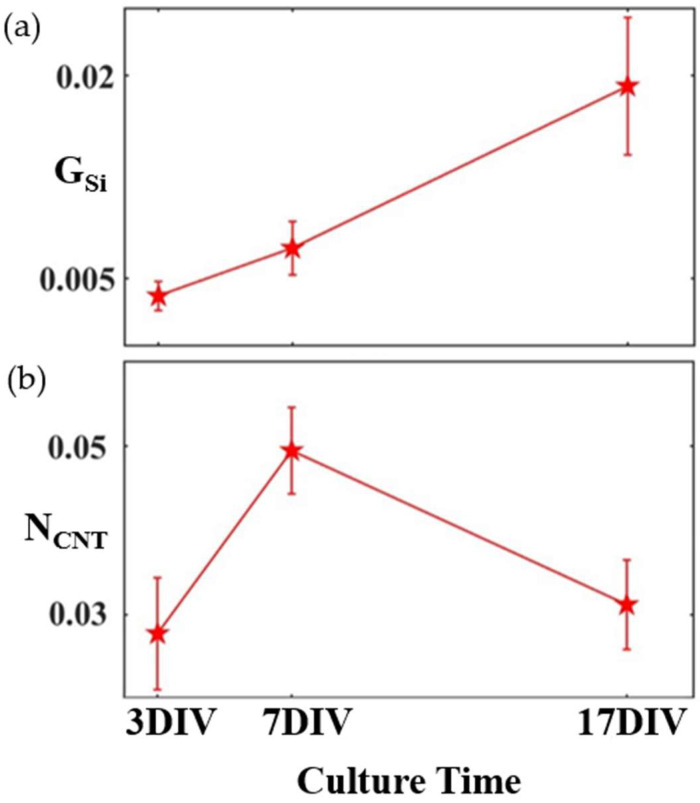
Time evolution of (**a**) *G_Si_* and (**b**) *N_CNT_* for electrodes made of rows of VACNTs separated by smooth areas of SiO_2_ averaged at three culture times [52]. The glial cells follow a gradual increase in surface coverage across the culture time, while the neuronal processes show a peak at 7 DIV. Error bars correspond to 95% confidence intervals.

While the principle of herding has been studied for various forest shapes (including both fractals and Euclidean patterns, such as grids) [14,50,51], Figure 1 shows previous measurements performed on rows of VACNTs [52]. The measure *G_Si_* quantifies the proportion of the SiO_2_ regions’ surface area covered by glial cells, and *N_CNT_* measures the total neuron process length on the electrode normalized to the electrode’s surface area (see Section 3). Between 3 DIV to 17 DIV, glia gradually accumulate on the smooth SiO_2_ surface, while *N_CNT_* shows a peak at 7 DIV, suggesting an initial period of establishing connectivity, followed by an optimization process. The aim of the current study is to use and exemplify *D* as an effective measure of a neuron’s geometry, specifically probing the neuron’s evolution during this optimization phase between 7 DIV and 17 DIV.

## 3. Materials and Methods

### 3.1. Electrode Fabrication and Characterization

The fabrication methods for synthesizing the VACNT electrodes have been thoroughly detailed previously [52,53]. In summary, 2-inch silicon wafers with a 300 nm thermal oxide (SiO_2_) top layer were patterned using photolithography in a clean-room environment. Following the development of the photoresist, a 2–5 nm aluminum adhesive layer was thermally deposited, succeeded by an electron-beam deposition of a 3–5 nm iron catalyst layer.

The VACNTs were synthesized on these catalyst patterns via chemical vapor deposition (CVD) in a 2-inch quartz tube. During the 3 min growth period at 650 °C, a 2:1 mixture of ethylene (C_2_H_4_) and hydrogen (H_2_) (200 and 100 SCCM, respectively), along with a 600 SCCM flow of argon (Ar), was maintained. The electrodes were then stored in integrated circuit trays within a desiccator cabinet.

**Figure 2:**
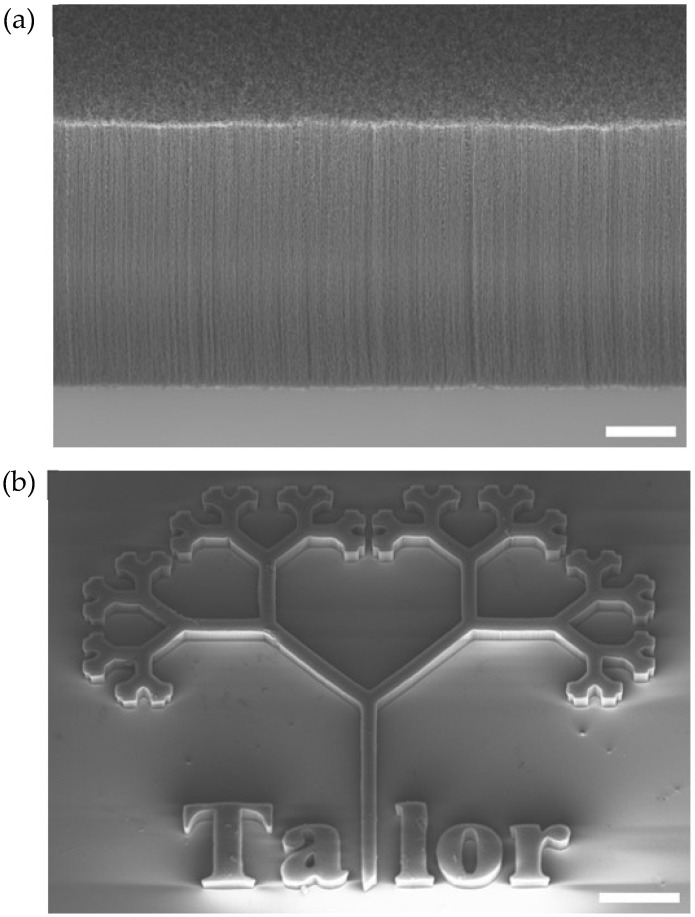
Scanning electron micrograph (SEM) images of patterned VACNT forests. (**a**) View of VACNT sidewalls taken at a 40° angle; scale bar corresponds to 10 μm; (**b**) top-down view of the lithographic patterning capability; scale bar corresponds to 250 μm.

Inspection of the top surfaces, sidewalls, heights, and general conditions of the VACNTs was conducted using a ZEISS-Ultra-55 scanning electron microscope (Figure 2). The heights of the VACNTs ranged from 16–36 µm. No precoating with poly-D-lysine (PDL) or poly-L-lysine (PLL) was used to enhance neuronal or glial adhesion to the various surface types. While the experiment presented in this study was conducted on square-shaped electrodes, Figure 2b showcases the patterning capabilities of the lithography technique described above.

### 3.2. Dissociated Retinal Cell Cultures

Dissociated retinal cell cultures were conducted as described in detail elsewhere [22,50]. In summary, wildtype C57BL/6J mice were housed at the University of Oregon (UO) animal welfare services, where they had continuous access to fresh water and food. All handling and experimental procedures followed protocols approved by the UO Institutional Animal Care and Use Committee (IACUC) under protocol 22-04, adhering to the National Institutes of Health guidelines for the care and use of experimental animals. Postnatal day 4 (PN4) mice were euthanized via decapitation, with their retinas being swiftly dissected and maintained in Dulbecco’s Modified Eagle Medium enriched with high glucose, sodium pyruvate, L-glutamine, and phenol red.

In each culture experiment, four retinas were placed in an enzyme solution of DMEM, papain, and L-cysteine. After enzymatic digestion, the retinas were rinsed with DMEM and then transferred to a fresh DMEM solution containing B27 and L-glutamine-penicillin-streptomycin. The dissociated retinal cells were centrifuged, and the cell pellet was resuspended in the DMEM/B27/antibiotic solution. The cells were cultured in 4-well plates, with 500 µL of the cell suspension and one sample per well. Cultures were maintained for 7 and 17 days in vitro (DIV) at 37 °C with 5% CO_2_. The culture medium was changed first at 3 DIV and then every other day until the end of the culture period.

### 3.3. Immunocytochemistry

The immunocytochemistry protocol has been described in detail previously [51,52]. In summary, the cells were fixed with 4% paraformaldehyde (PFA) and rinsed with phosphate-buffered saline (PBS). Then, the cells were pre-incubated in PBS-block. The PBS-block solution contained PBS, Triton-X, bovine serum albumin, goat normal serum, and donkey normal serum. The cells were then incubated overnight at 4 °C with PBS-block containing a mixture of two primary antibodies: mouse anti-β-tubulin III (neuronal marker [54,55]) and rabbit anti-glial fibrillary acidic protein (GFAP; glia marker). After rinsing, the cells were incubated with PBS-block containing two secondary antibodies: Cy3-labeled goat anti-mouse IgG and AlexaFluor-488-labeled donkey anti-rabbit IgG. Finally, the cells were rinsed with PBS, transferred to glass slides, and mounted with Vectashield containing DAPI.

### 3.4. Fluorescence Microscopy

**Figure 3:**
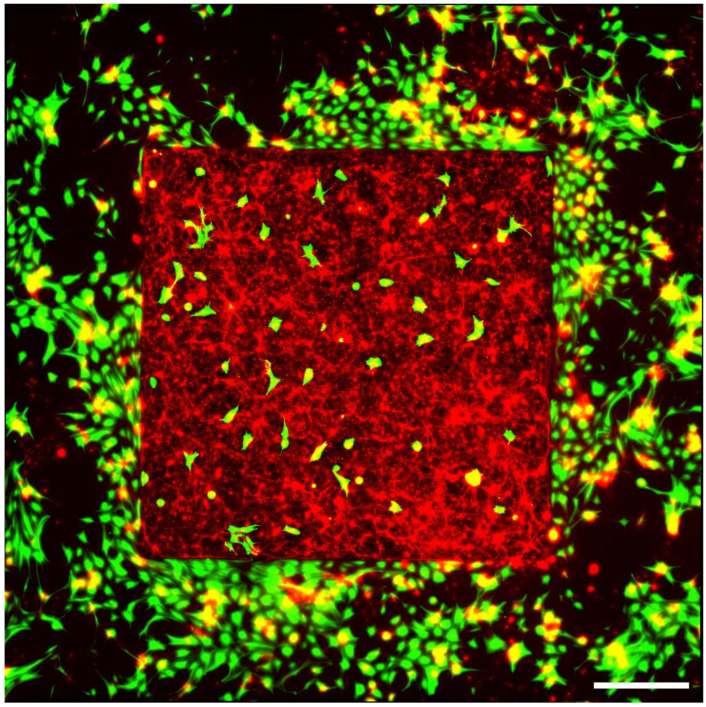
Fluorescence image of retinal cells interacting with a square VACNT electrode (with a side length of 2.145 mm) grown on a SiO_2_ substrate measured at 17 DIV (green = GFAP labeled glia; red = β-tubulin III labeled neurons). Scale bar corresponds to 500 μm.

A Leica DMi8 inverted fluorescence microscope was employed to capture 20× images of all electrodes in three channels: Cy3 (excitation at 550 nm with emission peak at 570 nm), AlexaFluor 488 (excitation at 493 nm with emission peak at 519 nm), and DAPI (excitation at 358 nm with emission peak at 461 nm). The imaging was split to focus separately on the VACNT top surface and the bottom SiO_2_ surface. Each field of view (FOV) was 2048 × 2048 pixels (662.65 × 662.65 µm^2^). Using an automated stitching algorithm, these FOVs were stitched together with a 10% edge overlap to produce complete images of the electrodes. Figure 3 shows a typical fluorescence microscope image for a square VACNT electrode.

### 3.5. Post-Culture SEM Imaging

Post-culture, cells were imaged using Scanning Electron Microscopy (SEM). Using 1.25% and 2.5% glutaraldehyde solutions in deionized (DI) water, the cells were fixed for 10 and 20 min, respectively. Following fixation, the wafers were rinsed three times in PBS, each for 10 min. For dehydration, the wafers were sequentially submerged in increasing concentrations of ethanol (50–100%) for 15 min each. Next, they were submerged in a 2:1 ethanol/HMDS solution for 20 min, followed by a 20 min rinse in a 1:2 ethanol/HMDS solution, and finally, a 20 min rinse in 99.9% HMDS. The cells were then left in fresh 99.9% HMDS overnight to allow evaporation. Before SEM imaging, the electrodes were coated with a 20 nm thick layer of gold.

**Figure 4:**
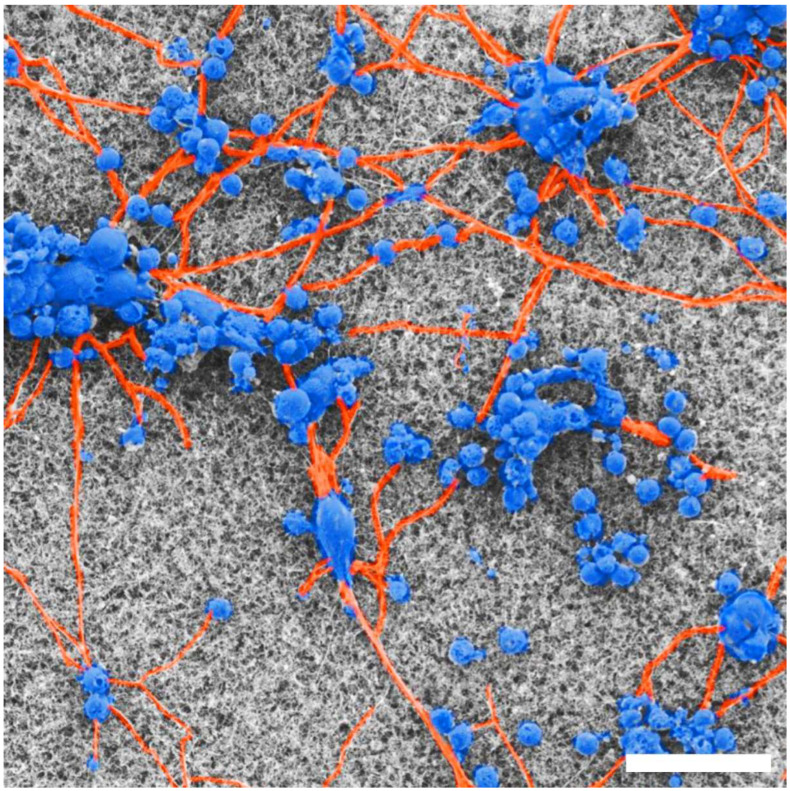
False-colored SEM image highlighting cell bodies (blue) and process (orange) behavior on the textured VACNT surface for 17 DIV. Scale bar corresponds to 25 μm.

Figure 4 includes false coloring of an SEM image to highlight the key properties of the neurons as they interact with the textured VACNT surface. The somas (blue) form clusters on both surfaces, connected via processes (orange), creating a network on the VACNT surface.

### 3.6. Alignment Analysis of Cultured Neurons

The primary goal of this experiment was the study of neurons’ growth in an unrestricted two-dimensional environment. In such an environment, we expected there to be no preferential growth direction of the neuron dendrites, whereas in patterned electrodes (such as rows), we did expect growth aligned with the pattern’s edges. As one measure to verify that neuron growth was, indeed, unrestricted upon the VACNT squares, an alignment analysis was performed using OrientationJ 2.0.4, a software plug-in for Fiji. Firstly, 20× magnification fluorescent images of β-tubulin III labeled neurons were stitched together to create a high-resolution image encapsulating the entire electrode. Secondly, these images were cropped to the center 1.8 mm side-length square of the electrode in an effort to mitigate any edge effects. Then, a mean orientation and coherency analysis is applied to this entire region, for each electrode presented for analysis. Briefly, the coherency describes the spread of orientations, where a measure of 1 indicates total alignment in one direction while a measure of 0 indicates a uniformly distributed alignment of neuron processes (i.e., no preferred or predominant orientation). In this context, a coherence value near 0 provides strong support that our square VACNT electrodes did, indeed, provide a platform for unrestricted growth.

### 3.7. Quantitative Measurement of Neuron Process Length N_CNT_

This analysis was conducted on the previously described fluorescence images of β-Tubulin III labeled neurons. The normalized process length on the VACNT (*N_CNT_*) surface was defined as the total process length across all FOVs (*N_LCNT_*) divided by the area of the electrode surface (*A_CNT_*). To minimize the error in detecting neuron process length near the electrode edges, the FOVs were manually inspected to ensure the correct detection of in-focus features.

### 3.8. Quantitative Measurement of Fractal Dimension D and Neuron Arbor Coverage S

As with the *N_CNT_* analysis, this analysis was based on the fluorescence images of β-Tubulin III labeled neurons. To analyze *D* and *S* (arbor coverage), it was first necessary to extract the dendritic arbor pattern of the individual neurons from the network of neurons formed on the electrode surface. The fluorescent images were first traced using a MATLAB R2023B edge-detection algorithm over a region encapsulating a particular soma of interest. These initial traces were refined employing a team of four observers to remove individual bias, who consistently employed a set of intuitive ‘rules’: (1) Dendrite processes are assumed to not ‘double back’ on themselves (based on a neuron’s need to make connections between neighbors in the neural network). This rule is absolute except in the rare case in which ‘doubling back’ is the only possible interpretation. (2) Neuron processes with directions greater than 90° angles relative to the radial lines emerging from the soma cannot originate from said soma unless no other interpretation is possible. (3) A process terminates when it reaches a neighboring soma. (4) In the case of processes connecting two somas, the originating soma is determined by visual brightness, thickness, and similarity to other processes originating from said somas (e.g., a process that tapers in width and brightness farther from a soma is determined to originate from it). After the application of these rules, traces were then extracted, skeletonized, and B/W binarized for further analysis. 

Twenty neuron arbor images were extracted using this manual procedure (ten each for 7 DIV and 17 DIV) from random positions on the electrode surface. The ten neurons analyzed for both 7 DIV and 17 DIV were sourced from three different electrodes each, for a total of six electrodes entering analysis. In each case, the neuron arbor trace was then covered with a mesh of identical squares (boxes). For an arbor to be considered fractal, the minimum number of boxes, *N(L)*, needed to cover the neuron arbor should scale with box size, *L*, as *N(L)~L*^−*D*^ [17,18,19,20]. The linearity of the plot indicates the neuron arbor is fractal, with a higher *D* fractal corresponding to a steeper slope and, thus, to the arbor pattern occupying more fine-scale boxes. Observation limits were set by coarse- and fine-scale ‘cut-offs.’ The fine scale limit was set approximately by the smallest feature size (the dendritic width), and the coarse scale limit was set approximately by the counting statistics of the box-counting algorithm (20% of the width of the arbor). For each neuron image, the spatial coverage *S* of the arbor was determined by measuring the enclosed area of its convex hull. Geometrically, a convex hull is the smallest convex polygon that encloses a set of points. In the context of individual neuron traces, it can be pictured with the analogy of the shape enclosed by an elastic membrane stretched over the end points of the arbor’s dendrites and serves as a robust and effective measure of the size of an arbor in the two-dimensional plane.

## 4. Results

**Figure 5:**
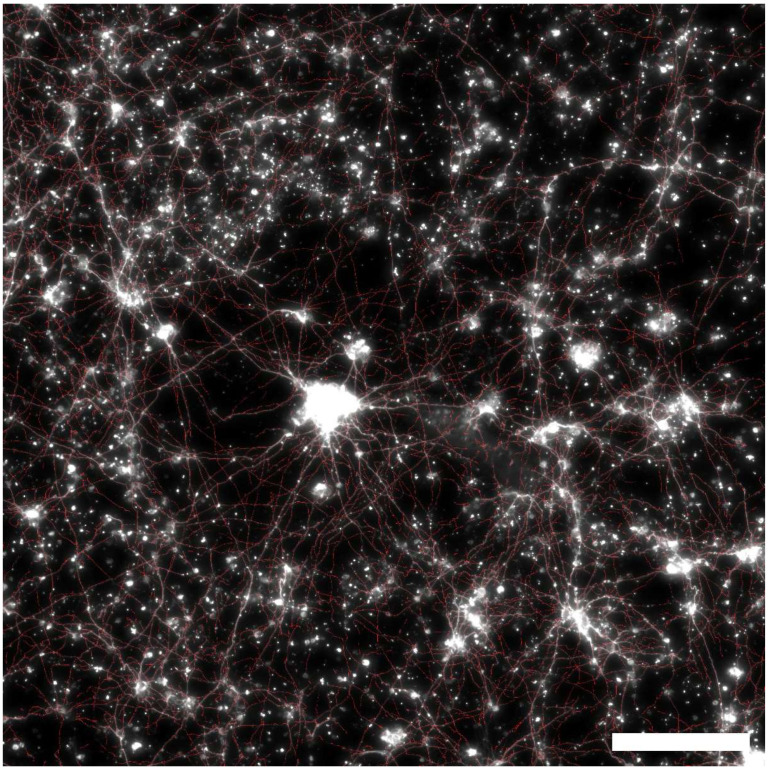
Representative fluorescence image of β-Tubulin III labeled neurons for 17 DIV superimposed on the neuronal processes identified by MATLAB tracing algorithm (red). Scale bar corresponds to 200 μm.

Figure 5 shows a representative fluorescence image of β-Tubulin III labeled neurons superimposed on the neuronal processes identified by the MATLAB algorithm (red) for a square-shaped electrode. The coherence measures for each electrode all indicated no predominant alignment of the neuron processes. For the three electrodes at 7 DIV and three electrodes at 17 DIV presented for analysis, the coherence values were, respectively, (0.005, 0.001, 0.004) and (0.010, 0.004, 0.004). This verifies that on the two-dimensional VACNT surface, the cultured neurons grew unrestricted. This provides a solid foundation for the rest of the analysis presented, as it ensures all following traces and analyses are performed on ‘naturally’ growing neurons, a key assumption in the process for creating bio-inspired electrode designs based on the fractal resonance hypothesis.

**Figure 6:**
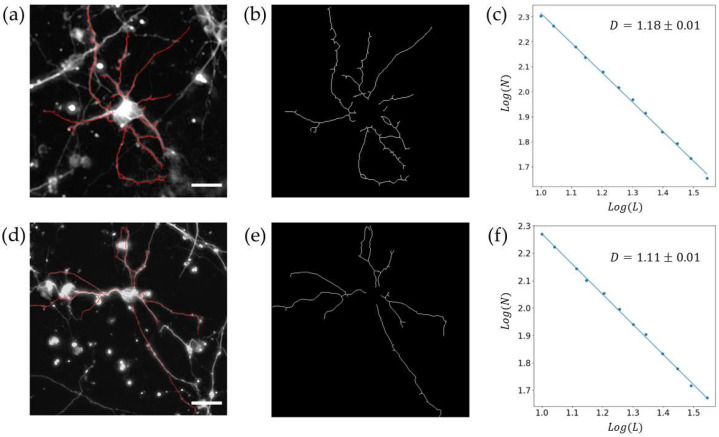
Fractal analysis of the individual neurons for 7 DIV (top row) and 17 DIV (bottom row). (**a**,**d**) Representative fluorescence images of β-Tubulin III labeled neurons superimposed on the manually extracted arbors (red); (**b**,**e**) the extracted arbors shown separately; (**c**,**f**) scaling plots generated when the box-counting algorithm is applied to the two arbor patterns shown in (**b**,**d**). The white size bars in (**a**,**d**) correspond to a distance of 25 μm.

**Figure 7:**
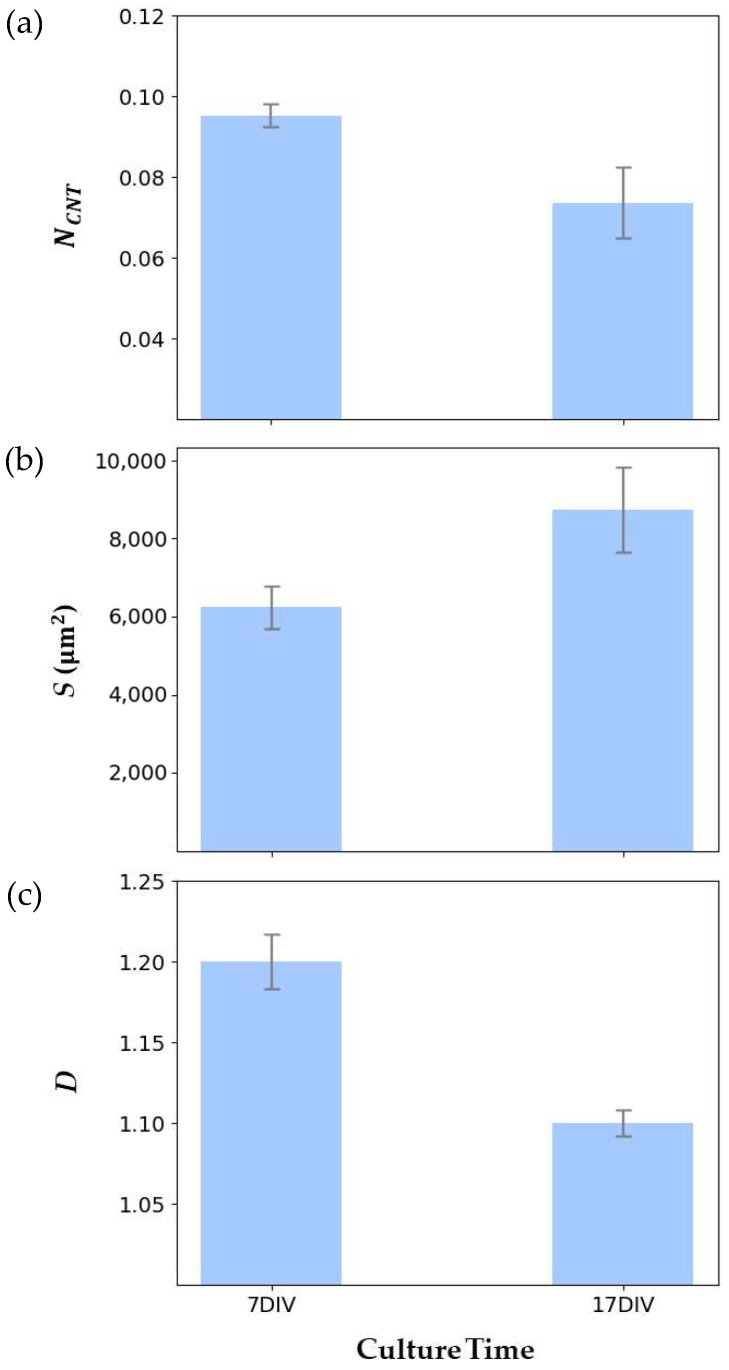
Quantitative analysis of the evolution of neuron characteristics measured from 7 DIV to 17 DIV for (**a**) *N_CNT_*, (**b**) *S*, and (**c**) *D*. In each case, the error bars correspond to standard error of the mean.

Figure 6 showcases traces of individual neurons at 7 DIV and 17 DIV from these networks, extracted by the manual procedure, superimposed on the arbor pattern of said neurons. The *N_CNT_* values measured at 7 DIV and 17 DIV are shown in Figure 7a, revealing a similar decrease to that observed previously for the VACNT rows in Figure 1. The mean values of *S* and *D* for ten individual neurons at 7 DIV and 17 DIV are presented in Figure 7b and Figure 7c, respectively. Although the individual arbors expand in spatial coverage over time, their *D* values decrease. A paired samples t-test was conducted on *N_CNT_*, *S*, and *D* to assess the statistical significance of the differences between 7 DIV and 17 DIV (see Table 1). The *N_CNT_* values for three electrodes each at 7 DIV and 17 DIV showed no statistically significant change, and the spatial coverage *S* for ten neurons each at 7 DIV and 17 DIV approached statistically significant change. In contrast, the differences in fractal dimension *D* were statistically significant between 7 DIV and 17 DIV, emphasizing *D* as a critical measure of neuronal network morphology.

## 5. Discussion

By measuring the *D* values of the neurons for two culture times, we have shown that their fractal character evolves in response to their interaction with the VACNT surface. In order to understand the observed decrease in *D*, we must examine the formation of neural networks in finer detail. Across culture time, neurons tend to aggregate into ‘clusters,’ as seen in Figure 4, gradually presenting structural characteristics similar to those of small-world networks. In small-world networks, each node (individual cells and clusters) connects to all other nodes through a small number of links (connecting neuronal processes), a configuration proposed to maximize efficient signal transmission [56,57]. In vitro research into small-world networks [58,59,60] describes this neural network formation as follows: Neurons begin by extending their processes in search of neighboring cells, reaching a state of maximum complexity featuring a large number of nodes and links between them. Following this, the neural network then starts to optimize when connection dynamics shift from governance from mostly neuron–substrate to also neuron–neuron interaction forces [61]. This process, and distinctly, the shift in connection dynamics, is consistent with the observations presented in this study—an initial increase in the total process length resulting in the maximal complexity state at around 7 DIV succeeded by evolution to an optimized state near 17 DIV (see Figure 1 and Figure 6). 

Figure 7 reveals that the arbors increase in spatial extent during the optimization process from 7 DIV to 17 DIV, indicating that the coarse-scale branches have increased. Visual inspections of the arbors (Figure 6b,e serve as illustrative examples) indicate that the fine-scale branches have also been pruned from the coarse branches. Recalling that the *D* value serves as a measure of the ratio of fine- to coarse-scale dendritic patterns, the increase in the coarse structure and decrease in the fine structure combine to reduce the *D* value. An interpretation of our results is that the neural network initially forms a high *D* network to quickly establish connections, but then optimizes by pruning small-scale dendrites to devote operational costs to the coarse branches. As an example of the costs, the metabolic expenditure of the neuron’s ion pumps increases with the surface area of the neurons’ dendrites [17,18,19,20], so pruning fine-scale branches will reduce these costs. 

## 6. Conclusions

The fractal resonance hypothesis for the interface between implant electrodes and neurons posits that optimal properties arise when the geometries of the two interacting surfaces are properly matched. By aligning the fractal dimensions of the implant and neurons, the goal is to foster unrestricted growth while maintaining a balance in connectivity cost—preserving optimal neuronal functions while the neural networks interact with an implant’s artificial surface. Favorable outcomes could include improved adhesion, growth, health, and ultimately, more effective electrical sensing and stimulation. Looking long-term, the practical aim and consequence of implementing fractal resonance is for each neuronal process to automatically seek out and grow along a fractal branch on the implant’s surface. This has the potential to induce selective growth, where neuron types with specific fractal dimensions can be specifically targeted to grow on implant branches with matching fractal dimensions. This specificity of interaction is particularly advantageous for implants needing to interact with networks comprised of multiple neuron types, enabling the organization of different neuron types in distinct regions of the implant. Based on these goals, the practical challenges of achieving fractal resonance lie in achieving two steps: (1) accurately measuring the *D* value of a particular type of neuron in its unrestricted state and (2) developing fabrication methods capable of producing electrodes that match the fractal dimension of neurons at an appropriate size scale. 

For step 1 in this procedure, in the current study, we imaged retinal neurons as they grew on the two-dimensional surfaces of VACNTs and detected an evolution in the *D* value. We conclude that it will be crucial to match the electrode shape to the optimized *D* value of the neurons that it interfaces with. This holds practical implications for retinal implants aimed at restoring vision to patients with degenerative retinal diseases [6,7,8,9,12,13,17,18]. Although conditions such as macular degeneration primarily impact the eye’s photoreceptors, they might also have an impact on the *D* value of the retina’s bipolar cells. If this is the case, then the electrode should be matched to the optimized state of the healthy neurons and not to that of the neuron’s diseased state. Indeed, the presence of the electrode might serve as a physical scaffold to re-grow the diseased neuron and restore its *D* value to its optimal operating structure. 

For step 2 of the procedure, the patterned electrodes should match the *D* values of the neurons. The choice to pattern the electrodes rather than simply use electrodes based on the two-dimensional planes of VACNTs stems from the additional favorable properties of adopting fractal branched electrodes compared to two-dimensional planes. These include (1) enhanced optical transmission, (2) enhanced mechanical flexibility, (3) larger electrical capacitances, and (4) the reduction of materials used to fabricate the electrode [62,63]. Although step 2 is not the focus of this paper, the image shown in Figure 2b highlights the potential of VACNTs for achieving fractal resonance. As mentioned in the Introduction, VACNTs have been proposed to have nano-scale textures that mimic some properties of the extracellular matrix, resulting in enhanced growth. Given the multi-scaled nature of this texture, a promising future approach would be to increase the texture amplitude to create three-dimensional structures with fractal patterns extending in both lateral and vertical directions. Figure 2 illustrates the patterning capabilities of the VACNT technique, showing that features can be patterned down to the micron scale. This capability, in principle, allows for the replication of the neuron patterns shown in Figure 6. To conduct step 2 in future fractal-resonance experiments, the aforementioned retinal neuron culture process would be repeated on the fractal electrodes in order to quantify the positive impact of fractal resonance on the neuron processes.

## Figures and Tables

**Table 1:** Quantitative analysis of the evolution of neuron characteristics measured from 7 DIV to 17 DIV for *N_CNT_*, *S*, and *D*. Values for 7 DIV and 17 DIV are means ± standard error of the mean.

Measure	7 DIV	17 DIV	*t*	*p*
*N_CNT_*	0.095 ± 0.003	0.074 ± 0.009	2.072	0.174
*S*	6240 ± 552.5 μm^2^	8750 ± 1088 μm^2^	2.031	0.072
*D*	1.20 ± 0.017	1.10 ± 0.008	4.586	0.001

## Data Availability

All experimental data within the article are available from the corresponding author upon reasonable request.
